# Effect of 24 weeks of intensification with a CCR5-antagonist on the decay of the HIV-1 latent reservoir

**DOI:** 10.1186/1758-2652-13-S3-O13

**Published:** 2010-11-04

**Authors:** L Díaz, C Gutiérrez, C Page, R Lorente, B Hernández-Novoa, A Vallejo, E Domínguez, M Abad, N Madrid, A Moreno, MJ Pérez-Elías, R Rubio, MA Muñoz-Fernández, S Moreno

**Affiliations:** 1 Immunobiology Department, Hospital General Universitario Universitario Gregorio Marañón, Madrid, Spain; 2 Infectious Diseases Service, Hospital Universitario Ramón y Cajal, Madrid, Spain; 3 Infectious Diseases Service Hospital Universitario Doce de Octubre, Madrid, Spain

## Background

It has been suggested that the stability of the CD4 cell reservoir could be related to the continuous replenishment from plasma-residual HIV-1. Intensification with an entry inhibitor could help eliminate the detectable levels of ongoing viral replication and accelerate the decay of the reservoir by inhibiting the entry of the virus into the cells.

## Methods

The intensification study with an entry inhibitor (maraviroc-MVC) included patients with stable antiretroviral therapy (ART) with three or more drugs, viral load (VL) of <50 copies/ml for two or more years, CD4 count of >350 cells/mm^3^ and CCR5-tropism. Latently infected resting CD4 cells were determined at baseline, week 12 and week 24, with further analyses planned at week 36 and week 48. Highly purified resting CD4 cells were isolated using magnetic beads rendering ~20 million and plated in replicate dilutions and stimulated in a limiting dilution culture assay, with irradiated PBMCs from a seronegative donor. Cells containing HIV-replication-competent were quantified by HIV-1 p24 Antigen Assay (results in infective units per million).

## Results

Nine patients have been included. Median time of HIV-1 diagnosis was 103 months [IQR 58-240], ART was administered for a median of 75 months [IQR 38-144], and the median baseline CD4 count was 711 cells/mm^3^ [IQR 547-793]. At baseline, the reservoir could be quantified in six out of nine patients. One patient has left the study, and the other eight patients continue with VL of <50 copies/ml and median CD4 count of 680 cells/mm^3^ [IQR 601-910]. After 24 weeks of intensification with MVC, when compared with baseline, a persistent decrease in the latent reservoir has been observed in three patients, no change (always undetectable) in two patients, and an increase, after an initial decrease, in the remaining three patients.

## Conclusions

Intensification with MVC seems to have a uniform, significant initial impact (measured at 12 weeks) on the HIV-1 latent reservoir that continues to be observed in most patients after 24 weeks.

